# Canakinumab treatment real world evidence in 3 monogenic periodic fever syndromes in 2009–2022: an interim analysis using the French JIR cohort database

**DOI:** 10.1186/s13075-024-03316-7

**Published:** 2024-04-08

**Authors:** Isabelle Koné-Paut, Sophie Georgin-Lavialle, Alexandre Belot, Magali Jover, Mathilde Pouriel, Laure Lacoin, Pascal Pillet, Véronique Hentgen

**Affiliations:** 1 Pediatric Rheumatology Department, APHP, Bicêtre Hospital, University of Paris Saclay, Kremlin Bicêtre, France; 2 CEREMAIA (French reference center for auto-inflammatory diseases and inflammatory amyloidosis), RITA network member, Paris, France; 3 Department of Internal Medicine, Sorbonne University, Tenon Hospital (APHP), Paris, France; 4 Pediatric Nephrology Rheumatology and Dermatology, CHU Lyon, Lyon, France; 5 RAISE (French reference center for inflammatory rheumatism and systemic autoimmune diseases in children), Paris, France; 6 Novartis Pharma, Rueil-Malmaison, France; 7 IT&M STATS, on behalf of Novartis, Neuilly-sur- Seine, France; 8 Epi-Fit, Bordeaux, France; 9 Pediatrics and Immunology, CHU Pellegrin, Bordeaux, France; 10 General Pediatrics, Versailles Hospital, Versailles, France; 11 CeRéMAIA – site constitutif CH Versailles Service de pédiatrie, 177, rue de Versailles, Le Chesnay, 78150 France

**Keywords:** Canakinumab, FMF, MKD, TRAPS, Treatment patterns, Safety, Real-world-evidence, JIR cohort, Autoinflammatory diseases

## Abstract

**Background:**

Our study aimed to provide real-world evidence on the treatment patterns, effectiveness and safety of canakinumab in France in Familial Mediterranean Fever (FMF), Mevalonate Kinase Deficiency (MKD), and Tumor necrosis factor Receptor Associated Periodic Syndrome (TRAPS).

**Methods:**

This study used the JIR cohort, a multicentre international registry created in 2013 to collect data on patients with juvenile inflammatory rheumatic diseases. French patients diagnosed with FMF, MKD or TRAPS and treated with canakinumab were included in this study.

**Results:**

31 FMF, 26 MKD and 7 TRAPS patients received canakinumab during the study period. Most of them initiated canakinumab at the recommended dose of 2 mg/kg or 150 mg, but less than half of FMF and MKD patients initiated it at the recommended frequency (every 4 weeks). Two years after initiation, the rate of patients still on treatment was 78.1% in FMF, 73.7% in MKD, and 85.7% in TRAPS patients. While the dose per injection remained globally the same over the course of the treatment, some adjustments of the dose intervals were observed. Six patients had a severe adverse event reported. Of those, three were possibly related to canakinumab.

**Conclusion:**

This interim analysis showed a good maintenance of canakinumab treatment 2 years after initiation and confirmed its safety profile in real-life practice in France in patients diagnosed with FMF, MKD and TRAPS. The high variety of dose and interval combinations observed in canakinumab treated patients let suppose that physicians adapt the posology to individual situations rather than a fixed treatment plan.

## Background

Familial Mediterranean Fever (FMF), Mevalonate Kinase Deficiency (MKD), Tumor necrosis factor Receptor Associated Periodic Syndrome (TRAPS), and Cryopyrin-Associated Periodic Syndrome (CAPS), comprise the most well-defined group of monogenic Auto-Inflammatory Diseases (AIDs). FMF is the most common form [1–3]. The onset of these diseases occurs often in infancy or childhood. They are characterized by recurrent episodes of fever of variable duration associated with signs of systemic inflammation. The skin, the digestive tract, and the musculoskeletal system are the main targeted sites of inflammation [4]. The presence of an increase of the C-reactive protein (CRP) during the attacks is frequent in this group of interleukin 1 (IL-1)-mediated diseases [4, 5].

The diagnosis relies on clinical features and may be confirmed by genetic testing revealing the presence of either pathogenic or likely pathogenic variant(s) in *MEFV* for FMF, *MVK* for MKD, *NLRP3* for CAPS, and *TNFRSF1A* for TRAPS [6].

Untreated Periodic Fever Syndromes deeply alter the patients’ quality of life. One of the major complications is the development of secondary amyloid A (AA) amyloidosis, which affects mainly the kidneys and may be life threatening.

Treatment aims to suppress systemic inflammation and to control disease activity. Minimizing subclinical inflammation between attacks is requisite for the prevention of disease related damage (in particular amyloidosis and renal failure), and for optimizing patient’s quality of life [7, 8].

As deregulated IL-1β secretion drives the systemic manifestations, IL-1 blockade is an effective treatment approach on systemic symptoms. Ilaris® (canakinumab) obtained a first European Marketing Authorization in the CAPS indication, and was marketed in France in August 2010. Several post-authorization studies have shown sustained efficacy using either anakinra or canakinumab in real life practice, with good tolerability and lasting improvement of quality of life [9–11]. In April 2018, canakinumab got approval in TRAPS, MKD and colchicine-resistant FMF (crFMF) indications following the results of the CLUSTER trial [3]. This phase III study included 181 adult, adolescent, and pediatric patients over 2 years old, diagnosed with TRAPS, MKD or crFMF. Patients received either canakinumab at a dose of 150 mg every 4 weeks (2 mg/kg if < 40 kg) or a placebo in the first treatment period. After week 16, the dose interval increased to every 8 weeks. Patients were followed up to 24 months. To reach disease control, the dosage of canakinumab was individually adapted, and results showed that required doses varied between individuals [12, 13].

Regarding FMF indication, daily oral colchicine is the core therapy and works in almost all patients when taken regularly and at a sufficient dose. However, around 5–10% of FMF patients have either insufficient responsiveness or intolerance to colchicine treatment (named colchicine-resistant FMF) [14]. Two IL-1 inhibitors, anakinra and canakinumab, are approved in crFMF in France. Since the effect of IL-1 inhibitors on amyloidosis is insufficiently established, IL-1 inhibitors are indicated in combination with colchicine in patients with crFMF.

In MKD and TRAPS indications, canakinumab is the only IL-1 blocker approved in France and is recommended as first-line maintenance therapy [8].

The use of canakinumab in real life in FMF, MKD and TRAPS may differ from the trial in terms of reasons for use, doses applied, and interval between injections. In addition, it is still unknown, especially in indications like crFMF if this treatment should be maintained lifelong. The aim of the present post-inscription study is to provide real-world evidence on the treatment patterns, effectiveness, and safety of canakinumab in a real-life setting in France in FMF, MKD, and TRAPS.

## Methods

All patients diagnosed with FMF, MKD, or TRAPS who initiated canakinumab in real life practice between January 1, 2009 and June 30, 2022 in the French JIR cohort centers participating to this study were included. We excluded patients treated in a clinical trial setting. Eight pediatric centers and three adult centers of the JIR cohort accepted to participate to this study at the time of our intermediate analysis.

The JIR cohort is an observational and multicenter international cohort created to collect data on patients with juvenile inflammatory rheumatic diseases (http://www.fondationres.org/fr/jircohorte - NTC02377245). Patients were enrolled after they (or their legal guardian) read the information document related to the JIR cohort project provided by their clinician and they confirmed that they were not opposed to the study and the storage of their personal data (non-opposition document completed and signed by the clinician). The French Ethics Committee (CCTIRS) approved the JIR cohort protocol on April 21, 2015 (decision number 14.302) and the National Commission of data processing and liberties (CNIL) approved the electronic form of the JIR cohort data collection on March 27, 2015 (decision number DR-2015-218).

In this study, the index date was the date of canakinumab initiation. Patients were followed until the cut-off date of interim data extraction, which took place on June 30, 2022. The diagnoses were those of the referring physicians, as well as the decision of canakinumab initiation. No definition of resistance to colchicine treatment in FMF patients was applied as there is still not enough consensus on a definition that could apply to each individual patient. Demographic data (age at diagnosis, genetics, and comorbidities), therapeutic data, data assessing disease activity and adverse events were extracted at baseline, and during the follow-up. Genotyping classification was based on the recommendations for genetic testing made by the International Study Group for Systemic Autoinflammatory Diseases [6]. Comorbidities of interest were AA amyloidosis, proteinuria, renal impairment, renal transplantation, chronic liver disease/cirrhosis, inflammatory bowel diseases, arthritis, ankylosing spondylitis, vasculitis, multiple sclerosis, coronary diseases, atherosclerosis and hematologic malignancies. Data evaluating disease activity included (i) CRP and serum amyloid A protein (SAA) levels, (ii) the Auto-Inflammatory Diseases Activity Index (AIDAI) score [15]. Therapeutic data included previous treatment(s) received, reason for canakinumab initiation, canakinumab posology (dose and frequency of injections), treatment duration, reasons for canakinumab discontinuation and time to re-initiation. Clinicians reported Adverse events (AEs) in their routine practice; the likelihood of canakinumab causality was based on clinician judgment only.

It was defined in the study protocol that analyses in subgroups with less than 5 patients would not be shown due to data protection reasons. Statistical analyses were performed using Python and EasyMedStat® software. Descriptive statistics were presented as median, 25th and 75th percentile (interquartile range (IQR)) for continuous variables and as numbers and percentages for categorical variables. The number and proportion of missing data for each variable were described. Treatment duration was measured using Kaplan-Meier method.

## Results

Up to June 2022, 692 FMF patients, 59 MKD patients, and 49 TRAPS patients were included in the JIR Cohort in France based on clinical diagnoses. The proportion of patients who received canakinumab was 4.8% of FMF patients (*n* = 33), 47.5% of MKD patient (*n* = 28), and 14.3% of TRAPS patients (*n* = 7). Two FMF patients and two MKD patients were excluded from this analysis as they received canakinumab in a clinical trial setting. The first patients treated with canakinumab in the JIR cohort initiated it in 2009, 2010 and 2015 in FMF, MKD and TRAPS indications respectively. 35.5% of FMF patients, 73.1% of MKD patients and 42.9% of TRAPS patients initiated canakinumab prior to the reimbursement in these indications in France (November 2017). The median follow-up in this interim analysis was 3.1 years (range: 0.2–12.0) for FMF patients, 4.6 years (range: 0.0-11.7) for MKD patients and 2.7 years (range: 0.5–6.2) for TRAPS patients.

### Characteristics of patients treated with canakinumab in real life practice

Patients’ characteristics are presented in Table 1. The median age at canakinumab initiation was 14.4 years in FMF patients (16 children (i.e. <18 years old) and 15 adults), 9.7 years in MKD patients (18 children and 8 adults), and 18.9 years in TRAPS patients (3 children and 4 adults). The proportion of female patients was of 80.7%, 65.4%, and 42.9% in FMF, MKD, and TRAPS cohorts respectively.

The median age at symptom onset was 3.0 years in FMF patients, 0.5 year in MKD patients, and 3.0 years in TRAPS patients. The median diagnostic delay was 1.3 years, 2.8 years, and 11.4 years in FMF, MKD, and TRAPS cohorts respectively.

In the FMF cohort, most of the patients (93.6%) carried homozygous mutation in the exon 10 of *MEFV* (M694I, M694V, and M680I). In the MKD cohort, 80.8% were homozygous or composite heterozygous for class 4 and 5 variants in the *MVK* gene. In the TRAPS cohort, 71.4% were heterozygous for class 4 and 5 variants in the *TNFRSF1A* gene.

Three FMF patients (9.7%) and one TRAPS patient (14.3%) had been diagnosed with AA amyloidosis prior to canakinumab initiation.

Among FMF, MKD, and TRAPS cohorts respectively, 48.4%, 53.8%, and 28.6% of the patients were naïve of biotherapy. On the contrary, 48.4%, 38.5% and 71.4% of them had received anakinra prior to canakinumab.

In the FMF cohort, most of the patients (90.3%) were on colchicine treatment, and 83.9% of them continued it after starting canakinumab. The median dose of colchicine was 1.5 mg/day prior to canakinumab initiation and 1.0 mg/day after canakinumab initiation. Patients without colchicine prior to canakinumab initiation were all receiving anakinra.

The lack of effectiveness of colchicine was the main reported reason for canakinumab initiation in biotherapy-naïve FMF patients (55.5%, 5/9). Canakinumab was initiated due to the lack of effectiveness of anakinra in almost half of the FMF patients switching from anakinra to canakinumab (46.1%, 6/13). Two patients switched from anakinra to canakinumab due to AEs.

In the MKD cohort, the main reported reasons for canakinumab initiation was the first line maintenance (52.6%, 10/19) and the lack of effectiveness of previous treatment (31.6%, 6/19). The impossibility to maintain previous treatment (40.0%, 2/5) was the main rationale in TRAPS patients.

**Table 1:** Baseline characteristics of patients initiating canakinumab in FMF, MKD and TRAPS

	Overall	< 18 years	≥ 18 years	Previously treated with anakinra	Not previously treated with anakinra	Patient treated after HTA approval
	n (%)	n (%)	n (%)	n (%)	n (%)	n (%)
**FMF**	*N* = 31	*N* = 16	*N* = 15	*N* = 15	*N* = 16	*N* = 20
**Demographic characteristics**						
Age (y), median (Q1-Q3)	14.4 (7.7–34.8)	7.7 (5.7–9.7)	37.1 (28.2–44.6)	14.4 (8.4–30.2)	15.6 (7.1–37.6)	16.7 (8.2–32.0)
Female	25 (80.6)	11 (68.7)	14 (93.3)	13 (86.7)	12 (75.0)	16 (80.0)
**Disease onset**						
Age (y) at first symptoms, median (Q1-Q3)	3.0 (2.3–4.2)	2.3 (1.0-3.3)	3.4 (3.0–9.0)	2.9 (1.1–4.5)	3.1 (2.6-4.0)	2.8 (2.4–4.1)
Time from first symptoms to diagnosis (y), median (Q1-Q3)	1.3 (0.4–2.8)	1.5 (0.7–2.1)	1.0 (0.3–6.1)	1.6 (1.1–3.7)	0.9 (0.2–2.1)	1.9 (0.7–3.6)
Time from diagnosis to canakinumab initiation (y), median (Q1-Q3)	7.3 (4.3–19.3)	4.7 (2.8–5.5)	21.0 (15.5–33.6)	8.9 (5.1–15.5)	6.9 (3.1–28.2)	8.1 (3.8–16.1)
**Genetic status**						
Confirmatory genotype: Homozygous mutations on *MEFV* Exon 10	29 (93.6)	15 (93.8)	14 (93.3)	15 (100.0)	14 (87.5)	20 (100.0)
Confirmatory genotype: Composite heterozygotes mutations on *MEFV* gene	2 (6.4)	1 (6.2)	1 (6.7)	0 (0.0)	2 (12.5)	0 (0.0)
**Complications/comorbidities**						
AA amyloidosis	3 (9.7)	0 (0.00)	3 (20.0)	3 (20.0)	0 (0.0)	1 (5.0)
Renal impairment	2 (6.4)	0 (0.00)	2 (13.3)	2 (13.3)	0 (0.00)	1 (5.0)
Renal transplantation	1 (3.2)	0 (0.00)	1 (6.7)	1 (6.7)	0 (0.00)	1 (5.0)
Ankylosing spondylitis	2 (6.4)	1 (6.2)	1 (6.7)	1 (6.7)	1 (6.2)	1 (5.0)
**Previous treatments**						
Colchicine*	28 (90.3)	15 (93.7)	13 (86.7)	12 (80.0)	16 (100.0)	18 (90.0)
Anakinra	15 (48.4)	8 (50.0)	7 (46.7)	15 (100.0)	0 (0.0)	9 (45.0)
Etanercept	1 (3.2)	1 (6.2)	0 (0.0)	0 (0.0)	1 (3.2)	0 (0.0)
**MKD**	*N* = 26	*N* = 18	*N* = 8	*N* = 10	*N* = 16	*N* = 7
**Demographic characteristics**						
Age (y), median (Q1-Q3)	9.7 (3.8–21.7)	6.2 (3.4–9.8)	24.1 (22.9–42.7)	7.5 (3.3–17.0)	12.4 (4.6–22.8)	9.5 (5.2–26.1)
Female	17 (65.4)	13 (72.2)	4 (50.0)	6 (60.0)	11 (68.7)	5 (71.4)
**Disease onset**						
Age (y) at first symptoms, median (Q1-Q3)	0.5 (0.25–1.23)	0.4 (0.2–0.9)	1.3 (0.4–3.8)	0.3 (0.3–1.3)	0.8 (0.2–1.2)	1.0 (0.3–1.2)
Time from first symptoms to diagnosis (y), median (Q1-Q3)	2.8 (1.8–7.8)	2.2 (1.4–3.8)	16.6 (11.1–24.8)	2.5 (1.3–7.5)	3.4 (2.1–8.5)	5.8 (2.0-17.8)
Time from diagnosis to canakinumab start (y), median (Q1-Q3)	2.2 (1.1–9.2)	1.8 (1.1–5.1)	9.1 (2.2–10.3)	2.2 (1.2–4.1)	3.6 (1.0-9.3)	1.6 (0.6–4.4)
**Genetic status**						
Confirmatory genotype: homozygous or composite heterozygotes with variant 4 and 5 on *MVK* gene	21 (80.8)	14 (77.8)	7 (87.5)	8 (80.0)	13 (81.3)	7 (100.0)
Non-confirmatory genotype: others mutations on *MVK* gene**	5 (19.2)	4 (22.2)	1 (12.5)	2 (20.0)	3 (18.7)	0 (0.0)
**Complications/comorbidities**						
AA amyloidosis	0 (0.0)	0 (0.0)	0 (0.0)	0 (0.0)	0 (0.0)	0 (0.0)
Renal impairment	0 (0.0)	0 (0.0)	0 (0.0)	0 (0.0)	0 (0.0)	0 (0.0)
Renal transplantation	0 (0.0)	0 (0.0)	0 (0.0)	0 (0.0)	0 (0.0)	0 (0.0)
**Previous treatments**						
Corticosteroid*	9 (34.6)	8 (44.4)	1 (12.5)	3 (30.0)	6 (37.5)	3 (42.9)
NSAIDs*	2 (7.7)	2 (11.1)	0 (0.0)	0 (0.0)	2 (12.5)	0 (0.0)
Anakinra	10 (38.5)	7 (38.9)	3 (37.5)	10 (100.0)	0 (0.0)	3 (42.9)
Etanercept	2 (7.7)	2 (11.1)	0 (0.0)	0 (0.0)	2 (12.5)	0 (0.0)
**TRAPS**	*N* = 7	*N* = 3	*N* = 4	*N* = 5	*N* = 2	*N* = 4
**Demographic characteristics**						
Age (y), median (Q1-Q3)	18.9 (15.1–38.2)	-	-	-	-	-
Female	3 (42.9)	-	-	-	-	-
**Disease characteristics**						
Age (y) at first symptoms, median (Q1-Q3)	3.0 (2.8-5.0)	-	-	-	-	-
Time from first symptoms to diagnosis (y), median (Q1-Q3)	11.4 (1.6–29.6)	-	-	-	-	-
Time from diagnosis to canakinumab start (y), median (Q1-Q3)	3.6 (2.2–9.1)	-	-	-	-	-
**Genetic status**						
Confirmatory genotype: heterozygotes with variant 4 and 5 on *TNFRSF1A* gene	5 (71.4)	-	-	-	-	-
Non-confirmatory genotype: others mutations on *TNFRSF1A* gene***	2 (28.6)	-	-	-	-	-
**Complications/comorbidities at canakinumab initiation**						
AA amyloidosis	1 (14.3)	-	-	-	-	-
Renal impairment	1 (14.3)	-	-	-	-	-
Renal transplantation	1 (14.3)	-	-	-	-	-
**Previous treatments**						
Corticosteroid*	1 (14.3)	-	-	-	-	-
NSAIDs*	0 (0.0)	-	-	-	-	-
DMARDs*	1 (14.3)	-	-	-	-	-
Anakinra	5 (71.4)	-	-	-	-	-
Etanercept	0 (0.0)	-	-	-	-	-

DMARDs: Disease-modifying antirheumatic drugs; FMF: Familial Mediterranean Fever, HTA: Heath technology assessment; MKD: Mevalonate Kinase Deficiency; NSAID: Non-steroidal anti-inflammatory drugs; TRAPS: Tumor necrosis factor Receptor Associated Periodic Syndrome

*Within 3 months prior to canakinumab initiation

**The other mutations identified in the 5 MKD with non-confirmatory genotype were the following: Val377Ile/Asp204Glu, Val377Ile/ Gly335Ala, Val377Ile/del 631-633GAG, Val377Ile/Gly335Ala and Val377Ile/wt

***The other mutations identified in the 2 TRAPS with non-confirmatory genotype were the following: Asp41Glu/wt and Arg121Gln/wt

### Canakinumab posology at the time of treatment initiation

The dose of canakinumab at initiation was missing for three FMF patients (1 adult and 2 children) and six MKD pediatric patients. Most of the patients in all three indications initiated canakinumab at the recommended dose of 2 mg/kg or 150 mg (85.7% of FMF, 65.0% of MKD, and 85.7% of TRAPS). All patients over 40 kg in FMF, MKD, and TRAPS indications started canakinumab with a dose of 150 mg per injection. More than half (58.3%) of the MKD patients and one-fifth (21.4%) of the FMF patients weighing less than 40 kg initiated canakinumab with a dose superior to 2.5 mg/kg (two with 2.5–3.5 mg/kg, 3 with 3.5–4.5 mg/kg and 5 with a dose of 150 mg).

The frequency of injections at initiation was broadly similar among FMF and MKD with less than half of the patients initiating canakinumab at the recommended dose interval of 4 weeks (45.2% and 34.6% respectively) and more than one-third initiating canakinumab every 8 weeks (35.5% and 38.5%). Two of the eight adult MKD patients initiated canakinumab with a dose interval inferior to 4 weeks. The proportion of patients initiated at the recommended dose interval of 4 weeks was slightly higher after November 2017 (65% of FMF and 42.9% of MKD). Among TRAPS patients, 57.1% (4 patients) initiated canakinumab every 4 weeks and 42.9% (3 patients) every 8 weeks. One FMF patient and one MKD patient were treated on demand.

### Canakinumab persistence and reason of discontinuation

Two years after canakinumab initiation, the rate of patients still treated was 78.1% (95%CI: 57.2–89.6) in FMF patients, 73.7% (50.5–87.2) in MKD patients, and 85.7% (33.4–97.9) in TRAPS patients.

Overall, 19 patients (8 FMF, 10 MKD, and 1 TRAPS) discontinued canakinumab during the study period. Of those, 13 discontinued it within the first 2 years following initiation (6 FMF, 6 MKD, and 1 TRAPS). Four patients (3 FMF and 1 TRAPS) (6.2%) discontinued canakinumab after the first injection: two due to AEs (respiratory tract infections) and two due to other reasons (weight gain and post-renal transplantation infection risk with IL-1 inhibitors). The reasons for discontinuation in the remaining FMF patients were remission (as reported by clinicians) for four patients and lack of effectiveness for one patient. The main reason for discontinuation in MKD patients was lack of effectiveness (80.0%, 8/10).

Three of the eight FMF patients and eight of the 10 MKD patients restarted canakinumab during the study period. Of the three FMF patients who restarted canakinumab, one restarted it less than one year after the initial discontinuation. The median time to restart canakinumab in MKD patients was 2.7 years.

### Evolution of posology during canakinumab treatment

While the dose of canakinumab per injection remained globally the same over the course of canakinumab treatment in all indications, we observed some adjustments of the dose intervals.

**Fig. 1:**
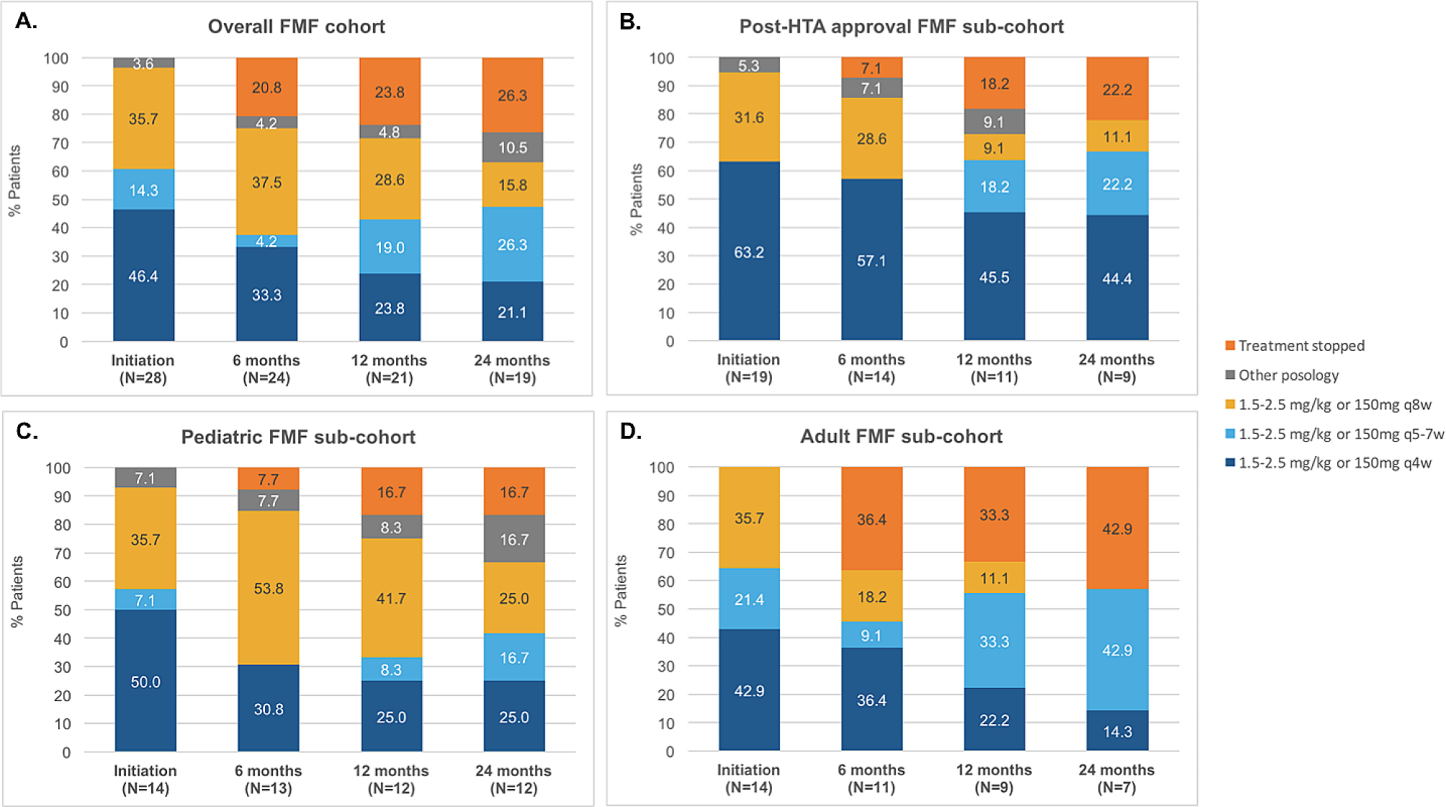
Evolution of canakinumab posology in FMF patients: overall (**A**), in patients who initiated canakinumab after health authority approval (**B**), in adult patients (**C**) and in pediatric patients (**D**). (FMF: Familial Mediterranean Fever, HTA: Heath technology assessment; q4w: every 4 weeks; q5-7w: every 5 to 7 weeks; q8w: every 8 weeks)

In the pediatric FMF sub-cohort, an increase of the dose intervals was observed at 6 months, 53.8% receiving canakinumab every 8 weeks at this point. It was followed by some adjustment of the dose interval with 25% of the patients having a reduction of the dose interval between 12 and 24 months. At 24 months, 25% were treated with 150 mg or 2 mg/kg every 4 weeks, 16.7% every 5–7 weeks, 25% every 8 weeks, 16.7% had another posology and 16.7% had already stopped canakinumab (including 1 patient due to remission). (Fig. 1)

In the adult FMF sub-cohort, no evolution of the dose intervals was observed in the first 6 months except for one patient who stopped due to remission. The same patient restarted treatment within the next 6 months. At 24 months, among the seven patients with sufficient follow-up, 14.3% received 150 mg or 2 mg/kg every 4 weeks, 42.9% every 5–7 weeks and 42.9% had already stopped canakinumab. (Fig. 1)

**Fig. 2:**
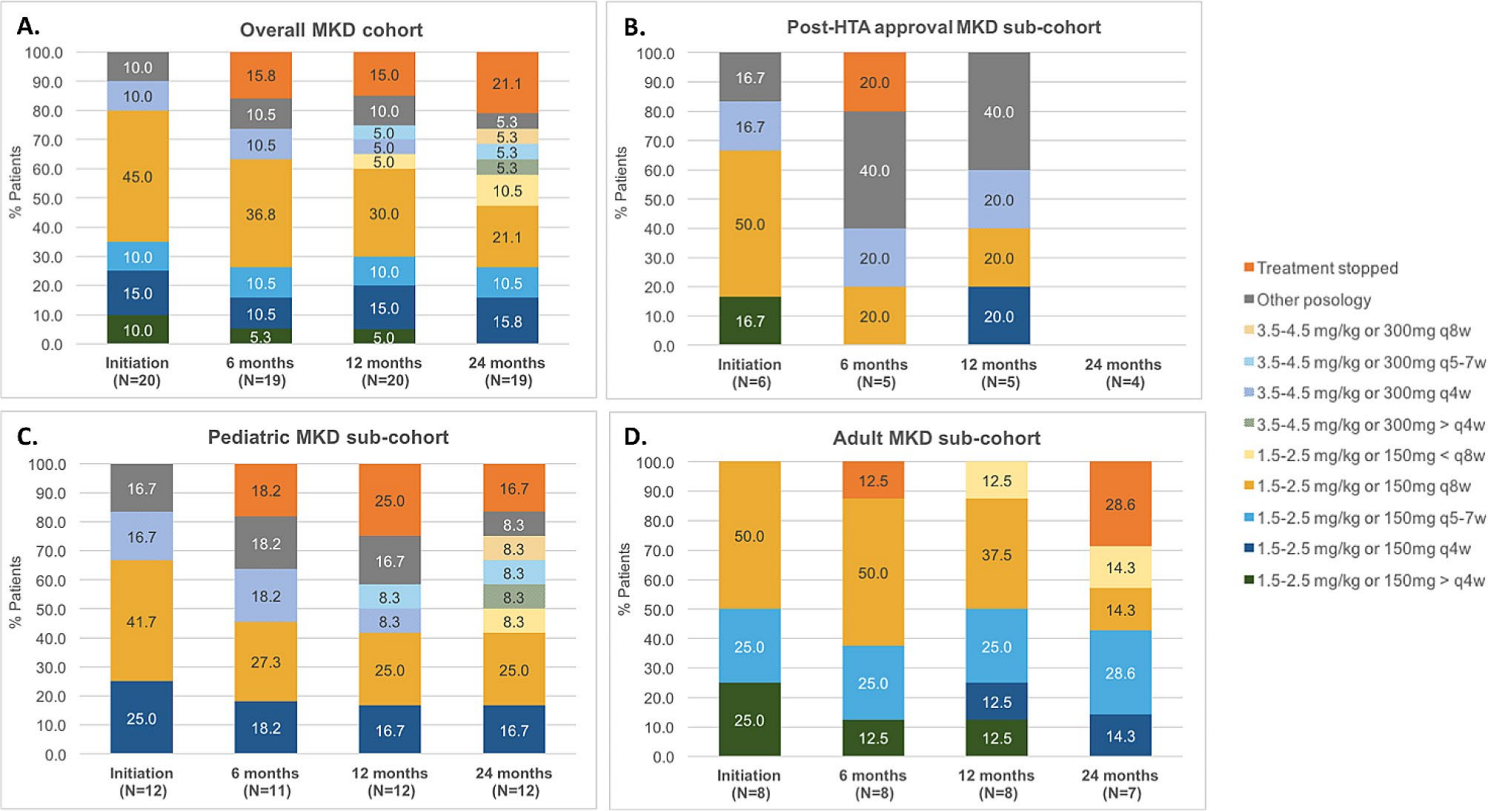
Evolution of canakinumab posology in MKD patients: overall (**A**), in patients who initiated canakinumab after health authority approval (**B**), in adult patients (**C**) and in pediatric patients (**D**). (HTA: Heath technology assessment; MKD: Mevalonate Kinase Deficiency; q4w: every 4 weeks; q5-7w: every 5 to 7 weeks; q8w: every 8 weeks)

In the pediatric and adult MKD sub-cohorts, no specific trends were identified in dose intervals evolution, but a wide range of dose and interval combinations were observed. At 24 months, among the 10 pediatric patients still on treatment, we observed seven different dose and interval combinations, which varied from 300 mg or 4 mg/kg per injection less than 4 weeks apart (highest posology observed) to 150 mg or 2 mg/kg per injection more than 8 weeks apart (lowest posology observed). At 24 months, among the 5 adult patients still on treatment, 4 different doses and interval combinations were observed from 150 mg or 2 mg/kg per injection every 4 weeks (highest posology observed) to 150 mg or 2 mg/kg per injection more than 8 weeks apart (lowest posology observed). (Fig. 2)

In the TRAPS cohort, at 12 months, two patients had canakinumab every 5–7 weeks, two every 8 weeks, one had another posology and one already stopped canakinumab.

### Evolution of colchicine dosing during canakinumab treatment in crFMF patients

Of the 26 of 31 FMF patients (83.9%) with concomitant colchicine at canakinumab initiation, six patients (23.1%) had a decrease in colchicine dose within the first two years of canakinumab initiation. None of the patients discontinued the colchicine during canakinumab treatment.

### Evolution of disease activity with canakinumab treatment

We had limited information on disease activity and inflammation biomarkers. Six months after canakinumab initiation, of the 12 patients with an AIDAI score (eight FMF and four MKD), 91.7% had a score inferior to nine (i.e. controlled disease). Among the 12 FMF patients and 12 MKD patients with a reported CRP at 6 months, 83.3% and 66.7% respectively had a CRP < 5 mg/l in an attack-free period. Among the 7 FMF and 4 MKD patients with a reported SAA at 6 months, 85.7% and 75.0% respectively had an SAA < 10 mg/l.

### Safety

Seven FMF patients reported nine AEs (one had three AEs), six MKD patients reported eight AEs (two had two AEs) and one TRAPS patient reported an adverse event. (Table 2). Four FMF patients, one MKD patient, and one TRAPS patient presented a severe AE. Of those, three were suspected to be related to canakinumab (all were infections, one requiring hospitalization or prolongation of hospitalization). Overall, of the 18 AEs reported, nine were infections and four were skin and subcutaneous tissue disorders. Two AEs in FMF, one in MKD, and one in TRAPS led to treatment discontinuation. Three AEs led to a dose reduction. None of the patients died during the study period.

**Table 2:** Reported adverse events during canakinumab treatment in FMF, MKD and TRAPS patients

	FMF (*N* = 31)	MKD (*N* = 26)	TRAPS (*N* = 7)
Total number of AEs reported during canakinumab	9	8	1
Patients with at least one AE	7 (22.6)	6 (23.1)	1 (14.3)
Number of AEs reported per patient			
0	24 (77.4)	20 (76.9)	6 (85.7)
1	6 (19.3)	4 (15.4)	1 (14.3)
2	0 (0.0)	2 (7.7)	0 (0.0)
3	1 (3.2)	0 (0.0)	0 (0.0)
At least one AE suspected to be related to canakinumab exposure	5 (16.1)	5 (19.2)	1 (14.3)
At least one serious AE* during canakinumab exposure	4 (12.9)	1 (3.8)	1 (14.3)
At least one serious AE* suspected to be related to canakinumab exposure	2 (6.4)	0 (0.0)	1 (14.3)
At least one AE leading to canakinumab discontinuation	2 (6.4)	1 (3.8)	1 (14.3)
Among reported AEs, types of AEs	*N* = 9	*N* = 8	*N* = 1
Infections	5 (55.5)	3 (37.5)	1 (100.0)
Skin and subcutaneous tissue disorders	2 (22.2)	2 (25.0)	0 (0.0)
Gastro-intestinal disorders	1 (11.1)	0 (0.0)	0 (0.0)
Nutrition disorders	1 (11.1)	0 (0.0)	0 (0.0)
Renal disorders	0 (0.0)	1 (12.5)	0 (0.0)
Lipid metabolism disorders	0 (0.0)	1 (12.5)	0 (0.0)
Nervous system disorders	0 (0.0)	1 (12.5)	0 (0.0)

AE: adverse event

*Serious AEs were defined as AEs which were life threatening, generated permanent disability, required an hospitalisation or a prolongation of the length of stay or could necessitate a medical or surgical intervention.

## Discussion

This study is the first describing the patterns of use of canakinumab in real-life setting in France in patients with FMF, MKD and TRAPS. Overall, our study showed high maintenance of canakinumab for the three indications but significant dosage adaptations, which ranged from a simple modification of dose, to a change in the interval between injections, and up to a suspension of treatment due to remission.

Most of the patient in the crFMF cohort had the most severe genotypes (93.5%). These genotypes lead to more severe disease (earlier onset, more frequent attacks, and higher risk of amyloidosis) [16–18] and therefore may increase the need for anti-IL-1 agents in this population as described in a previous study [19].

Most patients continued colchicine on canakinumab treatment as recommended, however five of them discontinued. Patients continuing colchicine with canakinumab received lower doses at initiation of canakinumab probably because, in this situation, colchicine was not supposed to prevent attacks anymore but rather to prevent secondary amyloidosis. Once the dose was fixed, at canakinumab initiation, we did not observe neither colchicine discontinuation nor dose reduction.

Although canakinumab is the only IL-1 inhibitor approved for the treatment of MKD and TRAPS, it did not appear systematically given to MKD and TRAPS patients in real-life practice: 47.5% of MKD patients, and 14.3% of TRAPS patients in the JIR cohort received canakinumab. This may reflect disease heterogeneity with possible low to mild phenotypes, management with off-label anakinra or hesitations of using costly treatments.

In all three indications, the recommended posology at canakinumab initiation is 150 mg every 4 weeks (or 2 mg/kg in patients < 40 kg) as mentioned in the summary of product characteristics approved by the European Medicines Agency (EMA). However, in this study, more than one-third of the patients initiated canakinumab at a frequency of one injection every 8 weeks. A potential explanation for this finding is that before the EMA approval for those three indications, the only approved use of canakinumab was for CAPS, and in this case, the dosing interval was every 8 weeks instead of 4 weeks. Therefore, in the absence of other guidance or evidence available, physicians followed the same scheme for FMF, MKD and TRAPS patients. Nevertheless, very little change in the treatment patterns at initiation occurred when restricting the analysis to patients who started canakinumab after 2017, date of reimbursement approval in France in those indications.

At initiation and over the course of canakinumab treatment, as described by Jeyaratnam et al. [10], younger patients with a body weight < 40 kg tended to receive a higher dosing regimen.

Multiple dose interval adjustments were made over the course of canakinumab treatment leading to a wide range of dose and interval combinations. Those results are in line with the long-term results of CLUSTER trial [12, 13] and several recently published data on canakinumab use in Eastern Europe [19–23]. To reach optimal control of disease, the posology of canakinumab could be adapted individually, by adjusting the dose or the frequency of injection. In this study, four FMF patients discontinued canakinumab due to remission which is consistent with recently published data in pediatric and adult populations [21–23]. Standardized recommendations regarding the extension of dose interval in case of complete remission under treatment are lacking. A recent publication (single-center study in Turkey) suggested a protocol to adapt canakinumab posology with an extension of dose interval in case a patient is in remission with the initial posology of 150 mg (or 2 mg/kg) every 4 weeks [23]. Clinical studies with longer follow-up and larger samples are needed to better understand the duration of treatment-free remission, the risk of relapse and the impact on quality of life.

Eight MKD patients discontinued canakinumab due to lack of effectiveness during the study period. However, from the available data, it is possible that these patients received an insufficient dose. Indeed, in the CLUSTER study patients with MKD tended to require higher doses of canakinumab than in other cohorts (FMF and TRAPS). In fact, the very high circulating levels of interleukin-1 in MKD patients are thought to be responsible for faster monoclonal antibody (canakinumab) depletion, which may justify increasing dosage over time in these patients. However, this gradual increase in dose is not seen in our study; long-term data would allow us to better test this hypothesis.

In line with the previous comment, most of those MKD patients restarted canakinumab during the study period.

Regarding canakinumab safety, our study confirmed the safety profile of canakinumab observed in clinical trials [3, 12, 13].

The strength of this study was to capture all patients treated with canakinumab in the participating centres. This allows to provide the full picture of canakinumab use in clinical practice including patients receiving only one dose.

Unfortunately, disease activity data and biomarkers were not carefully monitored in this retrospective cohort, which limits the correlation between the evolution of canakinumab posology and the control of the disease. Some indicators of the disease activity (notably the AIDAI score) did not exist for the patients included in the JIR cohort. We expect gathering more of these data in the next future.

Another limitation was the absence of information on the impact of canakinumab in the four patients with AA amyloidosis (three FMF and one TRAPS), as the subgroup analyses were restricted to subgroups of 5 patients or more due to data protection reasons.

## Conclusion

Overall, this interim analysis showed a good retention of canakinumab treatment and confirmed its safety profile in real life practice in France in patients diagnosed with crFMF, MKD and TRAPS. However, as a whole, canakinumab posology at initiation was lower than stated in the marketing authorization. A high variety of dose and interval combinations of canakinumab were observed over the course of the treatment, which let suppose that physicians adapt the posology to individual situations rather than a fixed treatment plan.

## Data Availability

The data that support the findings of this study are available from the JIR cohort database but restrictions apply to the availability of these data and so are not publicly available. Data are however available from the corresponding author upon reasonable request and with permission of the JIR cohort database.

## Abbreviations

AEs: Adverse Events
AIDAI: Auto-Inflammatory Diseases Activity Index
AIDs: Auto-Inflammatory Diseases
crFMF: colchicine-resistant Familial Mediterranean Fever
CRP: C-Reactive Protein
EMA: European Medicines Agency
FMF: Familial Mediterranean Fever
IQR: Interquartile Range
MKD: Mevalonate Kinase Deficiency
SAA: Serum Amyloid A protein
TRAPS: Tumor necrosis factor Receptor Associated Periodic Syndrome
